# Vulvo-Vaginitis in Children

**Published:** 1905-04-08

**Authors:** 


### VULVO-VAGINITIS IN CHILDREN.

That the mucous membrane of the vagina in childhood is extraordinarily susceptible to infection by the gonococcus is generally recognised, and all who have been engaged in the overseeing of a children's hospital are aware how rapidly and how mysteriously the contagion will spread on occasions, especially in the case of infants and very young girls. Dr. A. C. Cotton [1] relates the history of an epidemic in an American hospital in the course of which 19 children were involved. The diagnosis rests upon the presence of a purulent vulvar or vaginal discharge containing the intracellular gram-negative diplococcus of Neisser. Dr. Cotton suggests that the infection is spread either by improperly sterilised diapers or by the means of thermometers used to estimate the rectal temperatures in infants.

These discharges are very recalcitrant. In the author's series of cases the average duration was 116 days, and the most satisfactory treatment was found to be douching with 10 per cent. protargol; if this fails he suggests packing the vagina with swabs soaked in a 2 per cent. solution of the same drug, or, as recommended by Dr. Acker, in a 25 per cent. solution of ichthyol.

Fortunately these infections in early life are seldom marked by the remote lesions so common to the disease in later years. None of Dr. Cotton's cases developed an arthritis except one child, in whom the joint-lesion may have depended upon an attack of intercurrent scarlet fever, nor did any of them suffer from conjunctivitis of any severity. None the less, as was stated by Dr. Jacobi in the course of the discussion following the paper, not only does arthritis occasionally complicate the disease in childhood, but peritonitis, due to the dissemination of the gonococcus, is also liable to occur.

The moral of the epidemic, as drawn by Dr. Cotton, is that any case of purulent vulvo-vaginitis occurring in a children's hospital calls for rigorous isolation, not only of the patient, but of her nurse also, and of all utensils coming into contact with the infection. How far this ideal can be achieved in the service of the ordinary children's hospital we do not venture to say, though we have an opinion that it is a more drastic step than the circumstances demand. A disease whose *materies morbi* is so well identified and not, like the bacilli of diphtheria, inherently likely to be disseminated by such unpreventible actions on the part of the patient as coughing or crying, should be as capable of limitation by care in disinfection as is the infection of typhoid fever, and, like it, capable of being safely treated in a general ward. The essential matter is that all concerned should realise the extreme importance of unremitting attention to details of disinfection.

[1] Archives of Pediatrics, Feb. 1905.

